# Differential Antimicrobial Effect of Essential Oils and Their Main Components: Insights Based on the Cell Membrane and External Structure

**DOI:** 10.3390/membranes11060405

**Published:** 2021-05-28

**Authors:** Sergio Andrade-Ochoa, Karla Fabiola Chacón-Vargas, Luvia Enid Sánchez-Torres, Blanca Estela Rivera-Chavira, Benjamín Nogueda-Torres, Guadalupe Virginia Nevárez-Moorillón

**Affiliations:** 1 Facultad de Ciencias Químicas, Universidad Autónoma de Chihuahua, Circuito Universitario S/N, 31125 Chihuahua, Mexico; s.andrade.rat@gmail.com (S.A.-O.); kchacon@uach.mx (K.F.C.-V.); bchavira@uach.mx (B.E.R.-C.); 2 Escuela Nacional de Ciencias Biológicas, Instituto Politécnico Nacional, Prolongación de Carpio y Plan de Ayala S/N, Colonia Santo Tomas, 11340 Ciudad de Mexico, Mexico; luviasanchez@hotmail.com (L.E.S.-T.); bnogueda@gmail.com (B.N.-T.)

**Keywords:** essential oils, terpenoids, phenylpropanoids, cell membranes, antimicrobial, mechanism of action

## Abstract

The biological activity of essential oils and their major components is well documented. Essential oils such as oregano and cinnamon are known for their effect against bacteria, fungi, and even viruses. The mechanism of action is proposed to be related to membrane and external cell structures, including cell walls. This study aimed to evaluate the biological activity of seven essential oils and eight of their major components against Gram-negative and Gram-positive bacteria, filamentous fungi, and protozoans. The antimicrobial activity was evaluated by determination of the Minimal Inhibitory Concentration for *Bacillus cereus*, *Staphylococcus aureus*, *Listeria monocytogenes*, *Escherichia coli*, *Salmonella* Typhimurium, *Shigella sonnei*, *Aspergillus niger*, *Aspergillus ochraceus*, *Alternaria alternata,* and *Fusarium oxysporium*, the half-maximal inhibitory concentration (IC_50_) for *Trypanosoma cruzi* and *Leishmania mexicana*, and the median lethal dose (LD_50_) for *Giardia lamblia*. Results showed that oregano essential oil showed the best antibacterial activity (66–100 µg/mL), while cinnamon essential oil had the best fungicidal activity (66–116 µg/mL), and both showed excellent antiprotozoal activity (22–108 µg/mL). Regarding the major components, thymol and carvacrol were also good antimicrobials (23–200 µg/mL), and cinnamaldehyde was an antifungal compound (41–75 µg/mL). The major components were grouped according to their chemical structure as phenylpropanoids, terpenoids, and terpinenes. The statistical analysis of the grouped data demonstrated that protozoans were more susceptible to the essential oils, followed by fungi, Gram-positive bacteria, and Gram-negative bacteria. The analysis for the major components showed that the most resistant microbial group was fungi, which was followed by bacteria, and protozoans were also more susceptible. Principal Component Analysis for the essential oils demonstrated the relationship between the biological activity and the microbial group tested, with the first three components explaining 94.3% of the data variability. The chemical structure of the major components was also related to the biological activity presented against the microbial groups tested, where the three first principal components accounted for 91.9% of the variability. The external structures and the characteristics of the cell membranes in the different microbial groups are determinant for their susceptibility to essential oils and their major components

## 1. Introduction

Essential oils (EOs) are aromatic chemicals derived from plant material; they are complex mixtures of volatile secondary metabolites, which are water-insoluble [1,2]. They are generally liquid fluids, are lighter than water, have a strong and penetrating odor reminiscent of plant origin, and are colorless or yellowish translucent. Their functions are varied in plants: they are agents of pollination, reserve substances, part of the defense mechanisms against other plants (allelopathy) and certain insects, as well as healing and antimicrobial agents [3]. EOs are classified based on different criteria: the consistency, origin, and chemical nature of their main components.

EOs have been widely studied for their antimicrobial activity. For example, the EO of oregano has proven its effectiveness against a variety of microorganisms, including fungi such as *Candida albicans* and Gram-positive and Gram-negative bacteria [4,5]. Several authors have reported the broad antifungal activity of EOs against filamentous fungi, and their possible mechanism of action has been revised recently [6]. A recent report proposed an effect of thymol and salicylic acid on the membrane integrity and mitochondrial function of *Rhizopus stolonifer* [7]. Tea tree EO was reported to cause changes in mycelial morphology and membrane permeability of *Monilinia fructicola* [8]. There are also reports of their antiparasitic [9], insecticidal, and larvicidal activity [10,11].

The mechanism of action of EOs is not fully described due to their large number of components, and it is likely that their antimicrobial activity is not due to a specific mode of action but involves several targets in the cell [6,12]. It is believed that most EOs exert their antimicrobial activities by interfering with the cell membrane, with transport of electrons, ionic gradients, protein translocation, phosphorylation, and other enzyme-dependent reactions [13]. Some studies have shown that monoterpenes can interact with phospholipid membranes so that constituents of the EOs will act as interstitial impurities in the ordered structure of the lipid bilayer [14]. The terpenes, terpenoids, and phenylpropanoids, either alone or as part of EO, show different biological activities in different biological systems. In addition, the diversity of antimicrobial evaluation techniques provides an endless number of details that are difficult to correlate. The different experimental factors (methods for determining the activity, growth medium, and incubation conditions) can influence the antimicrobial activity of the EOs and their constituents [15]. In addition, most reports focus on the antimicrobial activity of the EO against a very homogeneous small group of microorganisms.

Among the plant spices most commonly used in Mexican cuisine are cinnamon, clove, cumin, and oregano, and some of their EOs have been reported to have antimicrobial activity [3,4,9]. In a previous report, we have demonstrated the larvicidal effect of these EOs and their major components [10], as well as their antimycobacterial action [15]. Therefore, we aimed to determine, by laboratory bioassays, the antibacterial, antifungal, and antiprotozoan activity of seven EOs and their major components. The EOs were obtained from species that were commercially available and included anise (*Pimpinella anisum* Linnaeus), cinnamon (*Cinnamomum verum* J. Presl), clove (*Syzygium aromaticum* (L.) Merr. & L. M. Perry), cumin (*Cuminum cyminum* Linnaeus), laurel (*Laurus nobilis* Linnaeus), Mexican lime (*Citrus aurantifolia* Swingle), and Mexican oregano (*Lippia berlandieri* Schauer).

The evaluation of the EOs and their major components for the different prokaryotic and eukaryotic organisms allowed the demonstration of differential effects based on their cellular membrane and external structures. This analysis can contribute to the ongoing discussion on the antimicrobial mechanisms of EO.

## 2. Materials and Methods

### 2.1. Extraction of Essential Oils and Characterization

Commercial spices were obtained from a local distributor (Commercial Cardona S. A., Chihuahua, Mexico), except for Mexican lime (local supermarket) and Mexican oregano, which was provided by CiRENA (Research Center for Natural Resources, Chihuahua, Mexico) [10,16]. Pure chemical compounds identified as major components of the EOs were acquired through Sigma-Aldrich (St. Louis, MI, USA). Essential oils were obtained using a modified Schilcher apparatus, using methodologies previously reported [10,16,17]. To determine the component present, EOs were analyzed with a Perkin Elmer AUTOSYSTEM XL Gas Chromatograph and TurboMass Gold Spectrometer (Perkin-Elmer, Norwalk, CT, USA) with a splitless injector and 70 eV electronic fragmentation detector. Each sample was injected on a non-polar PE-5 (5% phenyl-methyl-silicone, 60 m × 0.25 mm × 1 µm) column. Helium was used as a carrier gas with a flow rate of 1 mL/min. The detector and injector temperatures were maintained at 180 °C. The column temperature was maintained at 60 °C for 0.5 min with a 3 °C rise per min to 228 °C. Components were identified by their retention time and by comparison with the NIST library (NIST 02) based on the mass fragmentation pattern. For the MS analysis, a range of 35–430 *m*/*z* molecular weight was used, and the peaks were compared with their Kovats retention index (RI) [17].

### 2.2. Antibacterial Activity

Antibacterial activity was determined against *Bacillus cereus* (ATCC 11778), *Escherichia coli* (O157: H7 ATCC 43888), *Listeria monocytogenes* (clinical strain), *Shigella sonnei* (clinical strain), *Salmonella* Typhimurium (ATCC 14028), and *Staphylococcus aureus* (ATCC 25923).

The effect on cell metabolism for each EO and compound was evaluated by the Alamar Blue (Invitrogen, Carlsbad, CA, USA) reduction assay. The microbial strains were grown in Nutrient Agar (Bioxon, Mexico), incubated at 37 °C for 24 h; after incubation, a colony was transferred to a tube containing 4 mL of sterile phosphate buffer. It was homogenized using a vortex to adjust the amount of the 0.5 McFarland turbidity (1 × 10^8^ CFU/mL) and was further diluted (1:10) before use.

For determination of the Minimum Inhibitory Concentration (MIC), 200 µL of sterile deionized water in each of the wells (in a 96-well microplate) of the periphery was added to minimize dehydration during incubation except for the 1H, 2H, and 3H wells. To rows B through G in columns 2 through 10, 100 µL of EOs or the compound to be evaluated were added to different concentrations (1000, 750, 500, 250, 100, 75, 50, and 25 µg/mL) prepared in nutrient broth. Subsequently, we added the bacterial inoculum (100 µL). To row 11, we added 100 µL of nutrient broth and 100 µL of the inoculum to serve as a positive control. Then, we added 200 µL of nutrient broth to the 1H, 2H, and 3H wells to serve as negative controls and incubated the microplates at 37 °C for 24 h. Subsequently, we added 20 µL of Alamar Blue to the wells and allowed them to stand 6 h before determination of the MIC. A change in color from blue (oxidized state) to pink (reduced) indicated the growth of bacteria. We defined MIC as the lowest concentration of drug that prevented this change in color. Each reaction was carried out in triplicate.

### 2.3. Antifungal Activity

The antifungal activity was evaluated against *Aspergillus niger* (obtained from the collection of the Meritorious Autonomous University of Puebla collection), *Aspergillus ochraceus* (ATCC 22947), *Fusarium oxysporum* (obtained from the collection of University of the Americas in Puebla collection), and *Alternaria alternata* (isolated from a Jalapeño pepper). MIC was determined following the methodology described by Rasooli and Mirmostafa [18] and Rasooli et al. [19], with modifications.

Fungi were grown in Sabouraud medium by incubation for two weeks at 37 °C. Once the incubation time elapsed, we prepared a spore suspension by adding 20 mL of phosphate buffer (with Tween 80 at 0.5% *v*/*v*) to a Petri plate with fungal growth. Then, we moderately stirred the solution and allowed it to stand for 20 min. Then, we collected the buffer solution and counted the spores in a Neubauer chamber to determine the number of spores per mL.

To determine the MIC by microdilutions in a microplate, we added 100 µL of Sabouraud broth containing different concentrations of EOs or compounds to be evaluated (1250, 1100, 1000, 750, 500, 250, 100, 75, 50, and 25 µg/mL) to the wells. Subsequently, we added 5 × 10^5^ spores diluted in 100 µL Sabouraud broth to the wells for a final volume of 200 µL. Then, we incubated the plates at 28 °C for 48 h. MIC corresponded to the highest dilution at which the fungal strain did not show growth.

Peripheral wells were filled with 200 µL sterile distilled water to minimize dehydration of the medium. The 1H, 2H, and 3H wells served as negative controls, having within 200 µL of Sabouraud broth without spores or treatment. Then, we added 200 µL of medium and 5 × 10^5^ spores to the wells of column 11 as a positive control. We performed each biological evaluation in triplicate.

### 2.4. Antiparasitic Activity

The antiparasitic activity was evaluated against *Trypanosoma cruzi* (NINOA strain), *Leishmania mexicana* (ATCC MNYC-BZ/62/M379), and *Giardia lamblia* (WB strain).

The half-maximal inhibitory concentration (IC_50_) against *T. cruzi* (epimastigote) and *L. mexicana* (promastigote) was evaluated through the Alamar Blue (Invitrogen, Carlsbad, CA, USA) reduction assay. In both cases, the strains were maintained in RPMI 1× (In vitro Mexico City, Mexico) added with 1 mL of penicillin/streptomycin per 100 mL of medium. The medium was supplemented with 10 mL of fetal bovine serum (FBS SA Mexico in vitro) inactivated at 56 °C for 30 min.

The antitrypanosomal activity was evaluated using the Sykes criteria [20], with modifications. First, we cultured *T. cruzi* for 8 days in RPMI medium and then prepared a stock solution of epimastigotes 1.5 × 10^6^ parasites/mL. In a microplate, we added 200 µL of sterile distilled water to the wells in the periphery except for 1H, 2H, and 3H wells. Central wells were filled with 100 µL RPMI medium with different concentrations of EOs or compounds. Finally, we added 100 µL of the parasite suspension to each well (1.5 × 10^6^ epimastigotes/mL), leaving a final volume of 200 µL. To row 11, we incubated epimastigotes with 0.1% DMSO, which was used as control and as a positive control of metabolic inhibition. The 1H, 2H, and 3H wells served as negative controls.

*L. mexicana* was cultured 7 days in RPMI medium. In each well, 90 µL of RPMI medium containing 5 × 10^5^ promastigotes were added, which was followed by 10 µL of a stock solution of the EO or compound to be evaluated for a final volume of 100 µL. To row 11, promastigotes were incubated with 0.1% DMSO, which was used as control and as a positive control of metabolic inhibition. 1H, 2H, and 3H wells served as negative controls.

In both cases, microplates were incubated for 24 h at 27 °C in darkness. Alamar Blue was added (10% *v*/*v*) and plates were incubated for an additional 24 h period [21]. Data were obtained on a fluorometer, and IC_50_ was obtained using the Probit statistical tool [22].

*G. lamblia* was cultivated for 3 days in 13 × 100 mm borosilicate tubes with 5 mL of TYI-S-33 supplemented with 0.5 mL of serum, 0.05 mL of penicillin–streptomycin mixture, and 0.005 mL of bovine bile. A concentration of 2 × 10^5^ cells/mL of *G. lamblia* was used for each experiment, and the corresponding EO or the major component stock was added at different concentrations (500, 250, 100, 75, 50, and 25 µg/mL). The tubes were incubated at 37 °C for 24 h. After that, the tubes were cooled at 0–4 °C for 15 min, and the number of cells/mL was determined by microscopic count, using trypan blue staining and a Neubauer chamber. The lethal dose 50 (LD_50_) was calculated by Probit analysis [22]. Each biological evaluation was done in triplicate.

### 2.5. Statistic Analysis

The biological activity of the EOs and the pure major components was statistically analyzed by analysis of variance, considering the microorganism and the EO or the major components as fixed independent variables. Tukey’s mean analysis was carried out to determine differences among statistically different groups (*p* < 0.05). For evaluation of the biological activity, microorganisms were grouped by microbial groups, and major components were grouped by structure. In the overall analysis, the cross-interaction among the independent variables was included in the ANOVA. A Principal Component Analysis (PCA) was also carried out to evaluate the biological activity of EOs and major components to the microorganisms studied, taking into consideration the microbial groups and chemical structure of the major components as independent variables. The statistical analysis was done using the Minitab 20 Statistical Software (Minitab Inc., State College, PA, USA, 2021).

## 3. Results

### 3.1. Essential Oils Characterization

We used GC-MS to study the composition of the EO. The major chemical constituent of anise EO was identified as trans-anethole (88.9%) (Table 1) by comparison of mass spectral data, retention times (RT), and retention index (RI). The main constituents of cinnamon and clove EOs were cinnamaldehyde (73.3%) and eugenol (61.3%), respectively; for cumin, the main constituent was cuminaldehyde (41.3%), while for the laurel, it was eucalyptol (70.7%). Lemon EO presented limonene (92.3%) as a major compound, and oregano EO presented thymol (58.3%).

A total of 49 compounds were identified, with the EOs of cinnamon and cumin having the largest number of constituents. For the anise EO, only five compounds were found; however, these five components compromised 97% of the essential oil.

### 3.2. Antibacterial Activity

Seven EOs and eight pure compounds were evaluated against three Gram-positive bacteria and three Gram-negative bacteria. The results show that oregano EO has higher antibacterial activity for Gram-positive bacteria and Gram-negative bacteria, among which *Escherichia coli* and *Salmonella* Typhimurium are the most susceptible bacteria with an MIC value of 66 µg/mL. Thymol, the major compound of oregano EO, was the compound with the highest antibacterial activity, exerting its highest activity against *Staphylococcus aureus* with an MIC of 23 µg/mL. Carvacrol, an isomer of thymol, showed less antibacterial activity. The antibacterial activity of EOs and their major constituents are presented in Table 2.

In general, the EO with less antibacterial activity against Gram-positive bacteria was the EO of clove, while the EO of cumin was the one that presented the lowest activity against Gram-negative bacteria (600 µg/mL). Anethole was the pure compound with less antibacterial activity.

### 3.3. Antifungal Activity

Antifungal activity was evaluated against four filamentous fungi, which proved to be susceptible to EO of cinnamon and its main compound, cinnamaldehyde. At 66 µg/mL, cinnamon EO was able to inhibit the growth of *Aspergillus niger* and *Alternaria alternata*; cinnamaldehyde inhibited these microorganisms at 75 and 41 µg/mL, respectively. At 58 µg/mL concentration, cinnamaldehyde also inhibited *Aspergillus ochraceus* and *Fusarium oxysporum* (Table 3). EOs of laurel and lemon and their major constituents presented the lower antifungal activities.

### 3.4. Antiparasitic Activity

The most relevant antiparasitic activity against *Trypanosoma cruzi* and *Leishmania mexicana* was presented by cinnamon EO and cinnamaldehyde. Cinnamon EO presented an IC_50_ of 23 µg/mL against *T. cruzi* and 21 µg/mL against *L. mexicana*, while cinnamaldehyde showed activity at 10.4 and 8.6 µg/mL, respectively. On the other hand, oregano EO showed the highest activity against *Giardia lamblia* with an LD_50_ of 60 µg/mL, while thymol and carvacrol showed activity against this microorganism at 21.4 and 31.9 µg/mL, respectively (Table 4). EO of laurel and eucalyptol, its main compound, did not present relevant biological activity.

### 3.5. Analysis of Overall Biological Activity

To evaluate the overall effect of EOs and major components on the different organisms studied, they were grouped according to the characteristic of their external cell structures. The organisms were classified as Gram-negative bacteria, Gram-positive bacteria, filamentous fungi, and protozoans. The differences in the membrane and cell wall structure of bacteria are well characterized, with Gram-negative bacteria displaying an external cell membrane compared with Gram-positive bacteria. This difference has been related to their susceptibility to plant-derived antimicrobials [23].

The lime EO was not included for the statistical analysis, since it was tested only against the protozoan group. The analysis of the effect of the EOs on the microbial groups demonstrated a significant difference among them, where the lowest concentration of the EO for CMI/LC_50_/LD_50_ activity was considered with the most antimicrobial activity. The differences are as follows: Oregano > Cinnamon, Clove, Anise > Laurel, Cumin. 

**Figure 1:**
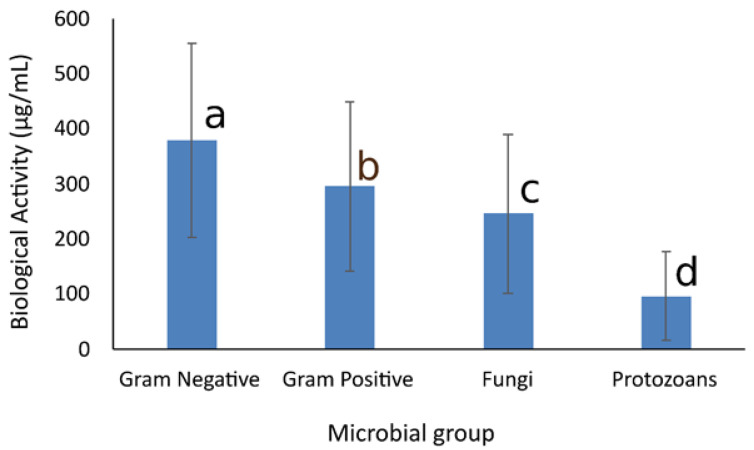
Overall antimicrobial activity of the essential oils (EO) on bacteria, fungi, and protozoans. The essential oils analyzed included anise (*Pimpinella anisum*), cinnamon (*Cinnamomum verum*), clove (*Syzygium aromaticum*), cumin (*Cuminum cyminum*), laurel (*Laurus nobilis*), and Mexican oregano (*Lippia berlandieri*). The biological activity for Gram-positive bacteria (*B. cereus, S. aureus, L. monocytogenes*), Gram-negative bacteria (*E. coli*, *S.* *typhimurium*, *S. sonnei*), and molds (*A. niger*, *A. ochraceus*, *A. alternata*, *F. oxysporium*) was CMI. IC50 was used for *T. cruzi* and *L. mexicana*, and LD50 was used for *G. lamblia*. The data represent the mean and standard deviation of 39 analysis. Differences among groups were established based on Tukey means analysis, following an ANOVA test.

The differences among the microbial groups are shown in Figure 1, where it is observed that the EOs are most effective against protozoans, followed by fungi and Gram-positive bacteria, while they are less effective against Gram-negative bacteria. As observed in Table 2, Table 3 and Table 4, the effect of each EO is different against the microorganisms studied, and this was also observed in the statistical analysis, with a highly significant statistical difference for the cross-interaction of EOs and microbial groups (data not shown).

Regarding the major components, the statistical analysis showed that there was a difference between the microbial groups and the compounds tested (data not shown). According to the proposed mechanisms of action of the main chemical components in EOs, the chemical structure and physicochemical properties are the predominant factors on their antimicrobial capacity.

**Figure 2:**
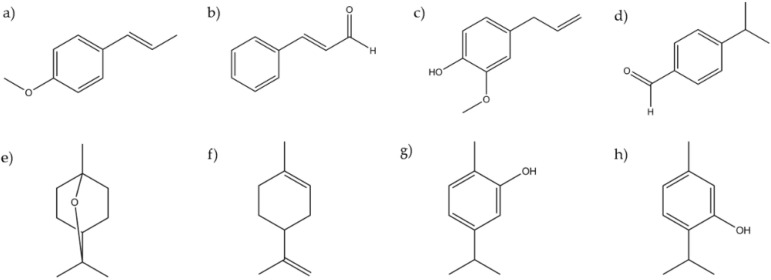
Chemical structures of the major components evaluated. (**a**) Anethole, (**b**) cinnamaldehyde, (**c**) eugenol, (**d**) cuminaldehyde, (**e**) eucalyptol, (**f**) limonene, (**g**) carvacrol, and (**h**) thymol.

Therefore, the chemical structure of the major components was identified as terpene (one compound included), phenylpropanoids, or terpenoids (Figure 2). Then, the chemical structure was used as a variable for the ANOVA analysis, and the results showed that the terpenoid and phenylpropane structures were more effective as antimicrobial compounds than the terpene. Regarding the microbial groups, they were separated based on their susceptibility to the compounds studied, as follows: Protozoan > Gram positive, Gram negative > Fungi.

**Figure 3:**
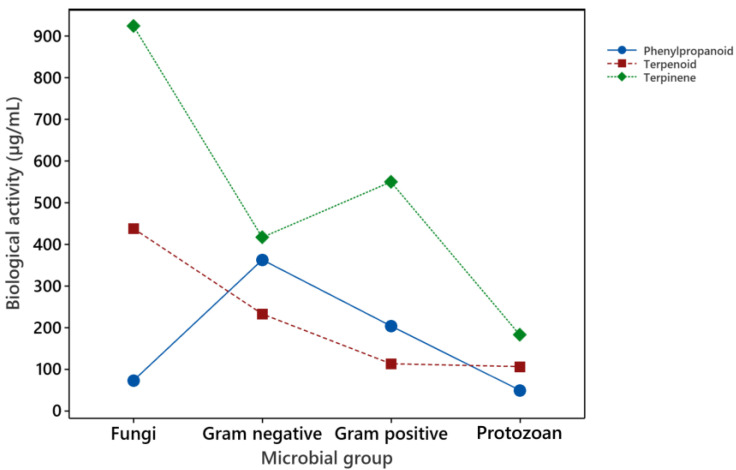
Interaction on the antimicrobial activity of major components of essential oils and bacteria, fungi, and protozoans. The chemical components were grouped according to their chemical structure in phenylpropanoids (cinnamaldehyde, eugenol, anethole), terpenoids (carvacrol, cuminaldehyde, eucalyptol, thymol) and terpinenes (limonene). The biological activity for Gram-positive bacteria (*B. cereus*, *S. aureus*, *L. monocytogenes*), Gram-negative bacteria (*E. coli*, *S.* *typhimurium*, *S. sonnei*), and molds (*A. niger*, *A. ochraceus*, *A. alternata*, *F. oxysporium*) was CMI. IC50 was used for *T. cruzi* and *L. mexicana*, and LD50 was used for *G. lamblia*. The points are the average of the measures for terpinenes (n = 12 for fungi, n = 9 for bacteria and protozoans), terpenoids (n = 48 for fungi, n = 36 for bacteria and protozoans), and phenylpropanoids (n = 36 for fungi, n = 27 for bacteria and protozoans).

Therefore, the most resistant microbial group to the chemical compounds studied was molds. In contrast with the EOs, fungi were more resistant to the pure components. It has been proposed that the mixture of components in the EOs can show synergistic effects, with differential activity as the pure compounds alone [24]. The differential effect of the compounds grouped according to their chemical structure against the microbial groups studied is presented in Figure 3, where it is observed that the phenylpropanoids are more effective against fungi and protozoans, and the terpenoids are more effective against bacteria and protozoans.

### 3.6. Principal Component Analysis of the Biological Activity

**Figure 4:**
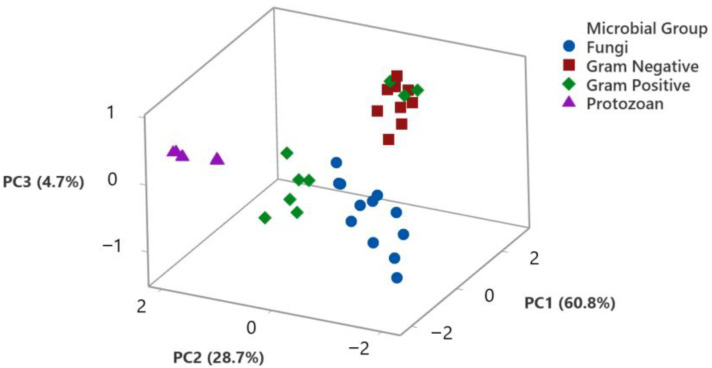
Principal Component Analysis (PCA) for the biological activity of essential oils against the different microorganisms analyzed. Scores for each microorganism for the first three PCA are shown.

**Figure 5:**
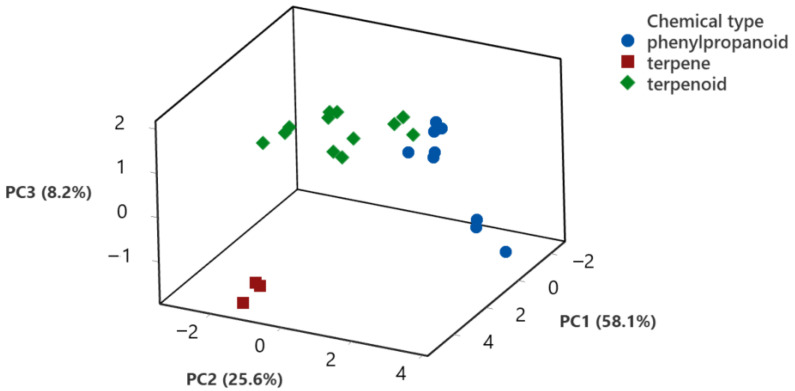
Principal Component Analysis (PCA) for the biological activity of major components of EOs against the different microorganisms analyzed. Scores for each chemical compound identified by their chemical structure for the first three PCA are shown.

To identify relationships between the biological activity of the EOs and their major components with the different groups of microorganisms included in this study, data were also analyzed by principal component analysis, as shown in Figure 4 and Figure 5.

The relationship of the biological activity of the different microorganisms, identified by their microbial group, with the first three components identified by PCA, is shown in Figure 4. The three first components accounted for 94.3% of data variation; PC1 explained 60.8% of the variation, while PC2 considered 28.7% and PC3 4.7% of the data variation. PC1 component has negative scores related to protozoans (−0.96 to −1.91 coefficient values), as well as for fungi (−0.59 to −2.18), while scores were positive for bacteria, with a higher positive load for Gram-negative bacteria (1.6 to 2.9). Regarding PC2, protozoans had a high positive and homogeneous load for this component (1.55 to 2.00), while fungi showed a negative load (−1.0 to −2.1) and bacteria has a wider score distribution (−0.8 to 1.6). Finally, PC3 had a positive load for protozoans (0.04 to 0.40). The analysis demonstrated that data were tightly grouped for anti-protozoan activity, while fungi were also grouped all together, and Gram-negative bacteria were also closely grouped.

In order to compare the effect of the major components present in the EOs studied regarding their biological activity, a PCA analysis using the chemical structure of the major components as an independent variable was also carried out. Figure 5 shows the scores for the first three components, considering the chemical structure of the compounds tested. The first three components accounted for 91.9% of data variability. PC1 explained 58.1% of variations, while PC2 explained 25.6% and PC3 8.2% of the total variation. Regarding the scores for the compounds, the terpene was separated from the other two groups, with a high positive load for PC1 (4.4 to 5.1 coefficient values) and negative loads for PC2 (−1.0 to −1.3) and PC3 (−1.4 to −1.7). Terpenoids had a wide distribution of scores for PC1 (−2.3 to 4.6) and PC2 (−3.0 to 1.4) and a positive load for PC3 (0.03 to 1.9). Finally, phenylpropanoids had a negative load in PC1 (−0.44 to −2.44) and a positive load in PC2 (0.14 to 3.6). PCA analysis was able to group the biological activity of the major components based on their chemical structure.

## 4. Discussion

The antimicrobial effect of EOs has been extensively documented, and there are many theories related to their mechanism of action, but many are related to their hydrophobic nature, which can then interact with the cell membrane [25]. The evidence of the destructive effect of EOs on the cell membrane includes the release of intracellular material when intact cells are placed in contact with the EO [26] or electron micrographs of microbial cells in interaction with EOs [27]. The determination of physicochemical functions by theoretical determination has also shown that the hydrophilicity of the chemical constituents of EOs is related to their biological activity [28]. Still, there is no consensus on the antimicrobial mechanism of action of the EOs or their chemical constituents. Scientific reports use different methodologies to measure antimicrobial activity; the chemical composition of the EOs or even the solvent used can affect the result. The essential oils used in this report have a composition similar to previous reports regarding the proportion of the major components [5,6].

The antimicrobial and antiparasitic activities are also in agreement with previous reports, where oregano and cinnamon EO and their major components are the most effective antimicrobials [5,29]. However, there are differences in an EO’s effect for each microbial strain tested, showing that the effect can vary depending on the microorganism, and there are even variations among strains of a similar microbial species [15]. In order to assess on a larger scale the differences in the effect of EO and major chemical constituents, we carried out a statistical analysis grouping the microorganisms studied, depending on their external structure. In addition, the chemical compounds analyzed were grouped according to their chemical structure in phenylpropanoids, terpinenes, and terpenoids. Since the method for testing antimicrobial activity was different, the biological activity was used to reference the effectiveness of EO and chemical compounds. For bacteria and fungi, the Minimal Inhibitory Concentration (MIC) was used; for *T. cruzi* and *L. mexicana*, the IC_50_ value was used, while LC_50_ was used for *G. lamblia*.

The overall effect of the EOs on the microbial group demonstrated that protozoans are more susceptible, which were followed by molds and bacteria; Gram-negative bacteria were the most resistant to the EO tested. The analysis suggests that the antimicrobial activity does not have a unique mechanism of action but is related to multiple actions on the target cell [23]. PCA can help with the identification of associations that are difficult to visualize at first sight. The graphic relationship of the biological activity of EOs vs. the microbial groups analyzed can be observed in Figure 4, where it is observed that the first three components generated by the analysis accounted for 93.4% of data variation. Protozoans were closely grouped, and fungal response to EOs was also grouped. The results are in accordance with the ANOVA results and demonstrate the differential effect of EOs on the microorganisms, which can be related to their external structures and differences in cellular membrane structure.

The toxic effect of EO and their terpenoid components on the structure and function of cellular membranes have been used to explain the antimicrobial action [26]. Some studies have demonstrated that the monoterpenes can interact with phosphatidic membranes, acting as interstitial impurities in the orderly structure of the lipidic bilayer [14]. Therefore, the hydrophobic nature of terpenoids is related to their attraction to cell membranes and cell walls [30]. If only the hydrophobic nature of EO components is responsible for their antimicrobial activity, it would be expected that bacteria would be more susceptible, given their positively charged cell wall. Even more, Gram-negative bacteria have a double membrane but are reported here and by many other authors to be more resistant to EO action [23]. It would also be expected for filamentous fungi to be more resistant given the presence of chitin and other carbohydrates in their cell wall [31]. In mycobacteria, the high hydrophobic nature of their cell wall is related to their in vitro susceptibility to EO [16]. Quantitative studies of the structure–activity relationship (QSAR) in mycobacteria have shown that the lipophilicity of terpenes, terpenoids, and phenylpropanoids is an important descriptor [32].

Fungi contain chitin mannoproteins and β-glucan in their cell wall chitin, providing a water-insoluble rigid structure, but it is not entirely hydrophobic. Fungal cell wall composition is partially repellent to EOs, but still, there are reports on damages to the cell membrane [7]. The antifungal activity of the EOs can be related more to the regulation of enzymes bounded to the cell membrane, which is responsible for the integrity of the fungal cell wall and not directly related to the interaction of EO with the membrane bilayer. Therefore, the hydrophobicity property of EOs becomes a secondary descriptor for the ligand–receptor interaction [33]. For example, trans-anethole has antifungal activity against the mold *Mucor mucedo*, showing hyphal morphological changes and an inhibition of chitin synthase activity in permeabilized hyphae in a dose-dependent manner [34]. It has been reported that some EOs or their components can inhibit ergosterol biosynthesis by interruption of the sterol normal biosynthetic pathways, generating osmotic and metabolic instability of the fungal cell, which compromises fungal reproduction and infectious activity [35].

Protozoans do not have a cell wall structure; therefore, their membranes are less permeable, but still, there are alterations on their cell membranes when exposed to EOs. For example, *Syzygium aromaticum* EO and eugenol have been reported to cause plasma membrane rupture and ventral and dorsal surface irregularities in *Giardia lamblia* trophozoites [27]. Treatment with *Ocimum canum* EO promotes ultrastructural alterations in the promastigotic forms of *L. amazonensis*, such as round, electron-dense corpuscles, which are characteristic of lipidic bodies, and dilation of the nuclear membrane, which shows discontinuity. In addition, an autophagosome-like structure with membranous and vesicular material and tubular structure was observed as well as changes associated with the depletion of ergosterol and the alteration of the physical properties of the membranes [36].

These observations suggest that EOs have as their main mechanism of action an effect on the protozoans’ cell membranes, but other mechanisms simultaneously occur. The question remains of whether those effects were the consequence of the initial damage to the cell membrane or the other way around. The determination of cell death markers by flow cytometry can provide information on the timeline of the events presented in the interaction of protozoans with EO or their major components. Flow cytometry analysis can suggest if the cellular damage to protozoans results from a necrotic or an apoptotic effect [29].

When the EO major components were analyzed, the data showed a statistical difference between the compounds and the tested organisms. To determine if the chemical nature of the components is related to their antimicrobial activity, the compounds were classified as terpenoids, terpinenes, and phenylpropanoids. In this case, fungi were more resistant to the chemical compounds tested, bacteria were equally susceptible, and again, protozoans were the most susceptible of the organism analyzed. The interaction between the microbial groups and the compounds grouped by chemical structure is presented in Figure 3, where it is observed that the phenylpropanoids were more effective against fungi and protozoans, while terpenoids were more effective against bacteria and protozoans. Phenylpropanes and terpenoids are proposed to directly affect the structure and function of the bacterial cell wall and cell membranes, and the results herein presented support this statement [37]. Regarding the effect on eukaryotic cells, the difference in the antifungal and antiprotozoal activity can be related more to enzyme-specific mechanisms of action than to direct interactions of the chemical components with cell wall or cell membrane structures. PCA analysis also demonstrated a difference in biological activity related to the chemical structure of the major components analyzed, as shown in Figure 5. The first three components generated accounted for 91.9% of data variation, with a clear association of phenylpropanoids and terpenoids. The information provides a good opportunity to apply in silico methodologies to identify possible therapeutic targets for the constituents of EOs. The extensive analysis of molecular coupling with various monoterpenoids and sesquiterpenoids with different and possible therapeutic targets of Leishmania [38] is the case of the extensive analysis of molecular coupling.

To suggest a unique mechanism of action for the chemical compounds present in EO is not suitable; evidence suggests that several targets participate in the inhibitory or microbicidal effect of the structural diversity of its constituent compounds. In fact, significant changes in the biological activity of structurally similar compounds can be observed. For example, thymol and carvacrol are isomers whose only structural change is the position of the hydroxyl in the phenolic group; this slight structural change implies relevant changes in their antimicrobial activity. The difference can be observed in their antibacterial activity, as presented in Table 2, Table 3 and Table 4. Using in vitro, density functional theory (DFT), and QSAR studies, our research group has shown that the presence of the hydroxyl group in the delocalized electro system is critical for the larvicidal, antibacterial, and antituberculosis activity of thymol and carvacrol [32,39,40]. The same effect can be observed in the different antimicrobial activities between anethole and eugenol, where the phenolic group has structural relevance, as observed by the antifungal and antiprotozoal activities of eugenol. On the other hand, the number of non-aromatic conjugated carbons (sp^2^) is relevant to the antimicrobial activity of cinnamaldehyde, which is a compound with relevant antiprotozoal, antifungal, and even antibacterial activity against Gram-positive bacteria.

These subtle structural differences between terpenoids and phenylpropanoids generate disparity between their antimicrobial activities and the diversity of observable cellular effects, leading us to believe that more than one mechanism of action is involved. It is even more complicated to suggest only one mechanism for EOs, which comprise a mixture of chemical compounds that present synergistic or antagonistic effects on their biological activity [25,33]. The effects of EOs are not the sum of the individual effects of their chemical components. In addition, multiple cellular targets in the cell will be affected by either the chemical compounds or the EOs. This work aimed to assess differences in the susceptibility to essential oils based on their cellular structure, considering that the antibacterial, antifungal, and antiprotozoal activity was tested under similar circumstances.

## 5. Conclusions

The antimicrobial activity of seven EOs and their major components was evaluated against Gram-positive bacteria, Gram-negative bacteria, and fungi by determining their CMI values. Antiprotozoan activity was tested by IC_50_ for *T. cruzi* and *L. mexicana*, and it was tested by LD_50_ for *G. lamblia*. Oregano and cinnamon EOs as well as their major components were the most effective. The overall activity was analyzed by ANOVA and PCA to test the effect of the EOs on the organisms by microbial groups; also, the major components were grouped according to their chemical structure. There was a relationship between the microbial groups and their sensitivity to EOs; protozoans were the most sensitive, followed by fungi, and the most resistant microbial group was Gram-negative bacteria. On the other hand, when the chemical nature of the major components was evaluated, differences were observed on the effect against fungi and bacteria. Membrane cellular composition and external structure can have a fundamental role in the response of the cells to EOs, but it is unlikely that a unique mechanism of action is responsible for the biological activity of EOs and their major components.

## Figures and Tables

**Table 1:** Chemical composition of the essential oils.

			Content %						
No.	RI	Compound	Anise	Cinnamon	Clove	Cumin	Laurel	Lime	Oregano
1	908	α-Thujene	-	-	-	0.2	-	-	-
2	934	α-Pinene	-	0.4	-	0.5	6.1	1.2	-
3	950	Camphene	-	0.5	-	-	-	-	-
4	964	Sabinene	-	-	-	-	8.1	-	-
5	979	β-Pinene	-	0.3	-	7.8	-	1.3	-
6	988	β-Myrcene	2.2	-	-	0.6	0.1	0.5	1.5
7	1009	3-Carene	-	0.2	-	0.1	-	-	-
8	1007	α-Phellandrene	-	1.1	-	0.1	-	-	-
9	1018	α-Terpinene	-	0.8	-	0.1	-	-	2.1
10	1031	Limonene	-	0.6	-	0.4	-	92.3	-
11	1032	β-Phellandrene	-	1.3	-	-	-	0.1	-
12	1027	p-Cymene	-	0.6	-	9.5	-	-	24.6
13	1087	α-Terpinolene	-	0.2	-	-	2.3	-	-
14	964	Benzaldehyde	-	1.2	-	-	-	-	-
15	1019	Eucalyptol	-	-	0.1	0.3	70.7	-	0.9
16	1055	γ-Terpinene	-	-	-	11.1	1.4	0.1	-
17	1088	α-Terpinolene	-	-	-	0.1	1.2	-	-
18	1102	Linalool	-	1.1	0.1	0.2	0.7	-	-
19	1130	Pulegone	-	-	-	0.1	-	-	-
20	1143	Terpineol-4	-	-	-	0.6	3.3	-	2.4
21	1160	Isopulegone	-	-	-	0.4	-	-	-
22	1176	Myrtenol	-	-	-	0.1	-	-	-
23	1196	Estragole	1.4	-	-	-	-	-	-
24	1200	p-Anisaldehyde	1.9	-	-	-	-	-	-
25	1228	Cuminaldehyde	-	-	-	41.3	-	-	-
26	1236	Chavicol	2.8	-	-	-	-	-	-
27	1255	Phellandral	-	-	-	0.2	-	-	-
28	1270	Safranal	-	-	-	1.9	-	-	-
29	1273	Thymol	-	-	-	-	-	-	58.3
30	1277	Carvacrol	-	-	-	-	-	-	3.4
31	1283	Cuminic alcohol	-	-	-	16.9	-	-	-
32	1295	trans-Anethole	88.9	-	-	-	-	-	-
33	1315	p-Mentha-1,4-dien-7-ol	-	-	-	0.3	-	-	-
34	1425	β-Caryophyllene	-	3.2	24.9	0.1	0.3	-	3.4
35	1463	Humulene	-	0.6	2.8	-	-	-	0.8
36	1200	α-Terpineol	-	0.5	-	-	-	1.1	-
37	1167	Hydrocinnamic aldehyde	-	0.2	-	-	-	-	-
38	1375	Hydrocinnamyl acetate	-	0.2	-	-	-	-	-
39	1591	Caryophyllene oxide	-	0.5	-	-	0.1	-	-
40	1279	Cinnamaldehyde	-	73.3	-	-	-	-	-
41	1453	Cinnamyl acetate	-	5.1	-	-	-	-	-
42	1357	Eugenol	-	2	61.3	-	0.3	-	-
43	1368	Methyleugenol	-	-	-	-	0.7	-	-
44	1313	Cinnamyl alcohol	-	0.3	-	-	-	-	-
45	1450	Cinnamic acid	-	0.1	-	-	-	-	-
46	1482	Eugenyl acetate	-	-	4.2	-	-	-	-
47	1491	β-Cadinene	-	-	3.6	-	-	-	-
48	1508	Epizonarene	-	-	0.1	-	-	-	-
49	1777	Benzyl benzoate	-	0.5	-	-	-	-	-
		Total	97.2	94.8	97.1	92.7	95.3	96.6	97.4
		Identified components	5	24	8	24	13	7	9

**Table 2:** Antibacterial activity of essential oils and their majority compounds.

Assays	MIC (µg/mL)					
	Gram Positive			Gram Negative		
Essential Oils	*B. cereus*	*S. aureus*	*L. monocytogenes*	*E. coli*	*S. typhimurium*	*S. sonnei*
Anise	383 ± 157.74 ^a^	316 ± 57.74 ^ab^	416 ± 57.74 ^b^	483 ± 28.87 ^ab^	483 ± 28.87 ^ab^	466 ± 28.87 ^ab^
Cinnamon	350 ± 0.00 ^ab^	283 ± 57.74 ^ab^	416 ± 57.74 ^b^	416 ± 57.74 ^bc^	416 ± 57.74 ^bc^	383 ± 57.74 ^bc^
Clove	283 ± 57.74 ^abc^	416 ± 157.74 ^a^	483 ± 28.87 ^b^	383 ± 57.74 ^bc^	383 ± 57.74 ^bc^	550 ± 86.60 ^ab^
Cumin	150 ± 86.60 ^cd^	200 ± 86.60 ^bc^	650 ± 0.00 ^a^	600 ± 86.60 ^a^	600 ± 86.60 ^a^	600 ± 86.60 ^a^
Laurel	216 ± 57.74 ^bcd^	216 ± 57.74 ^bc^	283 ± 57.74 ^c^	283 ± 57.74 ^c^	283 ± 57.74 ^c^	283 ± 57.74 ^c^
Lime	283 ± 57.74 ^ab^	216 ± 57.74 ^bc^	283 ± 57.74 ^c^	283 ± 57.74 ^c^	283 ± 57.74 ^c^	283 ± 57.74 ^c^
Oregano	83 ± 28.87 ^d^	83 ± 14.43 ^c^	100 ± 0.00 ^d^	66 ± 28.87 ^d^	66 ± 28.87 ^d^	100 ± 0.00 ^d^
Major Compounds	*B. cereus*	*S. aureus*	*L. monocytogenes*	*E. coli*	*S. typhimurium*	*S. sonnei*
Anethole	783± 57.24 ^a^	150 ± 86.60 ^a^	133 ± 28.87 ^d^	216 ± 57.74 ^b^	716 ± 57.74 ^a^	483 ± 28.87 ^a^
Cinamaldehyde	91 ± 14.43 ^c^	58 ± 14.43^b^	100 ± 0.00 ^d^	100 ± 0.00 ^c^	600 ± 86.60 ^ab^	283 ± 57.74 ^b^
Eugenol	250 ± 0.00 ^b^	50 ± 0.00 ^b^	216 ± 57.74 ^c^	133 ± 28.87 ^c^	416 ± 144.34 ^b^	316 ± 57.74 ^b^
Cuminaldehyde	116 ± 28.87 ^c^	41 ± 14.43 ^b^	91 ± 14.43 ^e^	50 ± 0.00 ^d^	316 ± 57.74 ^bc^	383 ± 57.74 ^ab^
Eucalyptol	83 ± 28.87 ^c^	141 ± 14.44 ^a^	416 ± 144.34 ^b^	600 ± 86.60 ^a^	666 ± 144.34 ^ab^	466 ± 28.87 ^a^
Limonene	750 ± 0.00 ^ab^	66 ± 14.43 ^b^	833 ± 144.34 ^a^	583 ± 144.34 ^a^	250 ± 0.00 ^c^	416 ± 57.74 ^a^
Carvacrol	91 ± 14.43 ^c^	23 ± 2.89 ^c^	200 ± 86.60 ^c^	83 ± 14.43 ^c^	41 ± 14.43 ^d^	83 ± 14.43 ^c^
Thymol	33 ± 14.43 ^d^	23 ± 2.89 ^c^	91 ± 14.43 ^e^	41 ± 14.43 ^d^	33 ± 14.43 ^d^	25 ± 0.00 ^d^

Each value is the average of a test conducted in triplicate. Superscripts correspond to groups by similarity analysis conducted by Tukey.

**Table 3:** Antifungal activity of essential oils and their majority compounds.

Assays	MIC (µg/mL)			
Essential Oils	*A. niger*	*A. ochraceus*	*A. alternata*	*F. oxysporum*
Anise	150 ± 86.60 ^bc^	150 ± 8.60 ^bc^	200 ± 86.60 ^bc^	150 ± 86.60 ^bc^
Cinnamon	66 ± 14.43 ^c^	83 ± 14.43 ^c^	66 ± 14.43 ^d^	116 ± 28.87 ^c^
Clove	150 ± 86.60 ^bc^	100 ± 0.00 ^c^	100 ± 0.00 ^cd^	116 ± 28.87 ^c^
Cumin	283 ± 57.74 ^b^	283 ± 57.74 ^b^	250 ± 57.74 ^b^	316 ± 57.74 ^b^
Laurel	483 ± 28.87 ^a^	500 ± 0.00 ^a^	450 ± 0.00 ^a^	450 ± 28.87 ^a^
Lime	316 ± 57.74 ^ab^	283 ± 57.74 ^b^	483 ± 28.87 ^a^	450 ± 28.87 ^a^
Oregano	250 ± 0.00 ^b^	283 ± 57.74 ^b^	200 ± 86.60 ^bc^	316 ± 57.74 ^b^
Major Compounds	*A. niger*	*A. ochraceus*	*A. alternata*	*F. oxysporum*
Anethole	750 ± 0.00 ^b^	483 ± 28.42 ^c^	500 ± 0.00 ^c^	650 ± 0.00 ^c^
Cinamaldehyde	75 ± 0.00 ^e^	58.3 ± 14.06 ^e^	41± 14.06 ^e^	58 ± 14.06 ^f^
Eugenol	100 ± 0.00 ^d^	66 ± 14.06 ^e^	41 ± 14.06 ^e^	66 ± 14.06 ^f^
Cuminaldehyde	283 ± 57.17 ^cd^	316 ± 57.17 ^cd^	66 ± 14 ^e^	91 ± 14.06 ^e^
Eucalyptol	716 ± 57.17 ^b^	683.3 ± 57.17 ^b^	716.6 ± 57.17 ^b^	716 ± 57.17 ^b^
Limonene	950 ± 0.00 ^a^	1000 ± 0.00 ^a^	750 ± 0.00 ^ab^	1000 ± 0.00 ^a^
Carvacrol	466 ± 28.42 ^bc^	466 ± 28.42 ^c^	783 ± 57.44 ^a^	616 ± 202.57 ^c^
Thymol	350 ± 0.00 ^c^	250 ± 0.00 ^d^	200 ± 86.23 ^d^	283 ± 57.44 ^d^

Each value is the average of a test conducted in triplicate. Superscripts correspond to groups by similarity analysis conducted by Tukey.

**Table 4:** Antiprotozoan activity of essential oils and their majority compounds.

Assays	IC_50_ (µg/mL)		LD_50_ (µg/mL)
Essential Oils	*T. cruzi*	*L. mexicana*	*G. lamblia*
Anise	52 ± 36.62 ^c^	63 ± 0.91 ^c^	136 ± 32.8 ^b^
Cinnamon	22 ± 0.22 ^f^	21 ± 25.13 ^e^	108 ± 22.53 ^e^
Clove	56 ± 7.88 ^d^	56 ± 8.35 ^c^	139 ± 11.35 ^bc^
Cumin	131 ± 115.53 ^e^	130 ± 19.02 ^d^	175 ± 43.62 ^d^
Laurel	223 ± 24.7 ^a^	209 ± 27.62 ^a^	193 ± 54.44 ^a^
Lime	98 ± 36.02 ^b^	88 ± 26.02 ^b^	112 ± 54.88 ^cd^
Oregano	23 ± 24.62 ^g^	59 ± 28.13 ^e^	60 ± 12.13 ^f^
Major Compounds	*T. cruzi*	*L. mexicana*	*G. lamblia*
Anethole	47.17 ± 5.90 ^d^	47.30 ± 2.32 ^d^	134.99 ± 0.65 ^b^
Cinamaldehyde	10.45 ± 0.19 ^g^	8.66 ± 2.18 ^f^	76.42 ± 2.79 ^e^
Eugenol	14.95 ± 1.11 ^f^	12.91 ± 12.29 ^ef^	104.04 ± 3.26 ^d^
Cuminaldehyde	65.92 ± 2.38 ^c^	55.96 ± 2.40 ^d^	141.16 ± 1.02 ^b^
Eucalyptol	275.43 ± 3.03 ^a^	262.09 ± 1.10 ^a^	265.43 ± 3.02 ^a^
Limonene	211.61± 1.63 ^b^	208.91 ± 3.13 ^b^	127.59 ± 316 ^c^
Carvacrol	36.43 ± 4.75 ^e^	79.77 ± 1.28 ^c^	31.92 ± 0.56 ^f^
Thymol	16.62 ± 1.71 ^f^	21.22 ± 1.30 ^e^	21.44 ± 52.07 ^g^

Each value is the average of a test conducted in triplicate. Superscripts correspond to groups by similarity analysis conducted by Tukey.

## Data Availability

Data are available from the authors upon request.
